# Does the Presence of Heavy Metals Influence the Gene Expression and Oxidative Stress in Bladder Cancer?

**DOI:** 10.1007/s12011-023-03950-3

**Published:** 2023-12-11

**Authors:** Eslam El-Agrody, Hassan Abol-Enein, Wael I. Mortada, Amira Awadalla, Heba H. Tarabay, Om-Ali Elkhawaga

**Affiliations:** 1 Department of Chemistry, Faculty of Science, Mansoura University, Biochemistry Division, Mansoura, Egypt; 2 Center of Excellence for Genome and Cancer Research, Urology and Nephrology Center, Mansoura University, Mansoura, 35516 Egypt; 3 Clinical Chemistry Laboratory, Urology and Nephrology Center, Mansoura University, Mansoura, 35516 Egypt

**Keywords:** Bladder cancer, Heavy metals, Oxidative stress, Gene expression

## Abstract

Heavy metal toxicity is associated with cancer progression. Studies have reported the relation between some metal ions and bladder cancer (BC). Direct influence of such agents in bladder carcinogenesis is still needed. Total 49 BC patients were included in the study. Level of Pb, Cr, Hg and Cd, oxidative stress markers, and gene expression of Bcl-2, Bax, IL-6, AKT, and P38 genes were detected in cancer and non-cancerous tissues obtained from bladder cancer patients. Concentrations of Pb, Cr, and Cd were significantly elevated in cancer tissues than normal, while Hg level was significantly increased in normal tissue than cancer. MDA level was significantly higher and SOD activity was lower in the cancer tissues compared to non-cancerous. The expressions of Bcl-2, IL-6, AKT, and P38 were significantly increased in the cancer tissues than in normal tissues while Bax level was significantly increased in non-cancerous tissue than in cancer tissue. In cancer tissue, there were significant correlations between Cr level with expression of Bax, AKT, and P38 while Cd level was significantly correlate with Bax, IL-6, AKT, and P38expression. The correlation between Cr and Cd with the expression of Bax, IL-6, AKT, and P38 may indicate a carcinogenic role of these metals on progression of bladder cancer.

## Introduction

Bladder cancer (BC) is the 10^th^ most commonly diagnosed cancer worldwide. It accounts for 3% of global cancer diagnoses [1] and 13^th^ cause of death [2]. The main types of BC are urothelial carcinoma or transitional cell carcinoma (TCC), squamous cell carcinoma (SCC), adenocarcinoma and small cell carcinoma. About > 95% of BC cases arise from cancer cells is the translational epithelium (urothelium) [3]. The exact cause of BC is unknown but the main risk factors of bladder cancer including: gender, age, smoking, obesity, diet, genetic Factors and environmental factors. The 2^nd^ risk factor of BC is occupational exposure which is responsible for about 18% of BC cases [1]. Approximately 25% of BC cases results from occupational exposure [4].

Exposure to carcinogens resulted from industries can cause deleterious health effect and increase risk of metal toxicity. Lead, cadmium and mercury are from the most metals that are responsible for human metal toxicity. These metals can enter human body through ingestion, inhaling contaminated dust or contaminated food supply [5]. Heavy metals can induce oxidative stress and bind to body proteins and enzymes that result in heavy metal toxicity [6].These metals have toxic effects on human health and could lead to cancer progression [7].

Several studies showed possible mechanism between these metals and progression of cancers [8] including bladder cancer [4, 9]. Cancer is a group of diseases characterized mainly by genetic mutations [10]. Bcl-2, Bax, IL-6, AKT, and P38 play a role in cancer progression.

Apoptosis is initiated by mitochondrial pathway and is regulated by proteins of the B-cell lymphoma-2 (Bcl-2) family to release apoptosis-activating factors. Pro-apoptotic members of Bcl-2 family (Bax) cause mitochondrial dysfunction. The apoptosis pathway is dependent mainly on the balance between the ratio of Bax and Bcl-2 genes expression [11].

IL-6 is a pleiotropic cytokine involved in immune responses, inflammation, and several processes [12]. And is activated in stress conditions such as ROS, microbial products, pro-inflammatory cytokines and UV. So, expression of IL-6 is associated with tissue damage [13].

AKT is a serine and threonine kinase that regulates cellular function by modulating the phosphorylation of various substrates which in turn plays an essential role in tumorigenesis. AKT has a key role in cell metabolism, proliferation and survival [14]. Akt pathway has been involved in cancer due to its critical role in cell survival and anti-apoptotic mechanisms. Many types of human cancer are associated with the upregulation of Akt, in some cancer types, Akt is associated with tumor aggressiveness [15].

P38 is one of the main subgroups of mitogen-activated protein kinases (MAPKs) which mediated cell proliferation, differentiation, death, migration, and invasion.P38 is activated in response to cellular and environmental stresses, inflammation, oxidative stress and other signal. P38 MAPK plays an important role in several human cancers including bladder cancer but the relation between the P38 MAPK pathway and tumor formation has is still not obvious [16].

To the best of our knowledge, no clinical reports have linked Pb, Cr, Hg, and Cd alterations with these previously mentioned genes in BC tissues. Therefore, the aim of this study was to determine whether concentrations of heavy metals such as Pb, Cr, Hg, and Cd have any effect on oxidative stress and expression of some genes Bcl-2, Bax, IL-6, AKT, and P38 genes in bladder cancer patients.

## Subjects and Methods

### Patients

The study included 49 BC patients admitted to Urology and Nephrology Center, Mansoura University, Egypt. All patients subjected to radical cystectomy. Cancer and non-cancerous tissue samples were obtained from each patient. Written informed consent was obtained from all patients. Institutional Review Board approval of Mansoura University, faculty of Medicine was obtained for this study (MS.21.12.1795). the participants have no known exposure route to heavy metals or other toxin.

## Methods

### Determination of Heavy Metals

From each BC patient, two tissue samples were collected, one from the central part of the tumor (cancer tissue), the second from the non-cancerous area of the bladder (non-cancerous tissue). Tissue samples digestion were performed as following: tissue samples (weighing from 100 to 300 mg) were placed in digestion vessels, 4 ml HNO_3_and 2 ml H_2_O_2_ were added to each vessel and incubatedfor15 min. The vessels were heated in a microwave oven (Speedwave four, Berghof Products, Germany) using a one-stage digestion program as follows: 1600 W (100%); 15-min ramp; at 200 °C temperature; 15-min hold; and 15-min cooling After cooling, the resulting solution was diluted to 10.0 ml with double distilled water [17]. The obtained solutions were subjected to Pb, Cr, Hg and Cd analysis using inductively coupled plasma optical emission spectrometry (Agilent technologies 720 ICP-OES Series, Santa Clara, CA, USA).

### Determination of MDA Level and SOD Activity

Bladder tissues were weighed and washed by phosphate buffered saline (PBS), pH 7.4, homogenized with 0.1 M phosphate buffer (pH 7.4) then centrifuged at 1,500 × *g* at 4 °C, the supernatants were collected and stored at − 80 °C until analysis. MDA level and SOD activity were assessed using commercially available test kits (Biodiagnostics, Cairo, Egypt) [18].

### Quantitative Reverse Transcription PCR Reaction

Bladder tissue samples were obtained from each patient and stored at − 80 °C in RNA later (AM7024, Invitrogen Corporation, Grand Island, NY, USA). Total RNA has been extracted from tissue using Trizol kits (Invitrogen Corporation, Grand Island, NY, USA) following the manufacturer’s protocol. The concentration and integrity of RNA samples were measured by the Thermo Scientific NanoDrop 2000c spectrophotometer (NanoDrop Technologies, Wilmington, USA) and the agarose gel electrophoresis stained with ethidium bromide, respectively. RNA samples were converted to complementary DNA (cDNA) with High Capacities cDNa reverse transcription Kit (Thermo Fisher Scientific, Waltham, MA, USA). cDNA sampled stored at − 80 °C. Quantitative RT-PCRwas performed using SYPER Green PCR Master Mix (Thermo Fisher Scientific, Waltham, MA, USA). The mRNA expression level of Bcl-2, Bax, IL-6, AKT, and P38 as well as GAPDH as a housekeeping gene (internal control) were quantified using Step one plus real-time PCR (Applied Biosystems). Primers sequences of studied genes were listed in Table 1. The PCR cycle parameters are adapted on the basis of the next programmer: pre-denaturation step for 10 min at 95 °C, 40 cycles in denaturation step for 15 s at 95 °C, annealing step for 1 min at 60 °C and finally, extension for 1 min at 72 °C. The relative quantification was calculated using equation RQ = 2 − ΔΔCT [19].

**Table 1:** List of primer sequence

Gene	Sequence	product length (bp)	Accession no
Bcl2	F: 5- GTGGAGGAGCTCTTCAGGGA-3	304	XM_047437733.1
	F: 5- AGGCACCCAGGGTGATGCAA-3		
Bax	F: 5- GGCCCACCAGCTCTGAGCAGA-3	527	XM_047439168.1
	F: 5- GCCACGTGGGCGTCCCAAAGT-3		
IL-6	F: 5- TACATCCTCGACGGCATCTC-3	466	NM_001371096.1
	F: 5- GCTACATTTGCCGAAGAGCC-3		
AKT	F: 5- ACCTTTTGCGGCACACCTGA-3	120	NM_001382431.1
	R:5- CAGGCGACCGCACATCATCT-3		
P38	F: 5- GCATAATGGCCGAGCTGTTG -3	130	NM_001315.3
	R: 5- TCATGGCTTGGCATCCTGTT -3		
GABDH	F: 5- GTCTCCTCTGACTTCAACAGCG -3	131	NM_001357943.2
	R: 5- ACCACCCTGTTGCTGTAGCCAA -3		

### Immunohistochemistry

Bladder cancer tissues were cut into 4μm sections, heated overnight at 37 °C, and deparaffinized with xylene. Polyclonal antibodies of anti-apoptotic Bcl2 (Cat no: 60–0005-7, Genemed Biotechnologies, Inc.) and Apoptotic Bax (Cat no. A5-11,378, Thermo scientific, USA) were used in the immunoperoxidase approach. The expression of Bcl2 and Baxwere detected at 1:100 dilutions. Immunostainingwas performed using Power-Stain™ 1.0 Poly HRPAEC Kit (Cat. No. 54–0022) using 3.3′-diaminobenzidine (DAB) as a chromogen. Poly HRP conjugate was added for 30 min, the sections were washed with PBS, and DAB chromagen staining was applied to develop the reaction color. The tissue sections were counterstained with hematoxylin, dehydrated, mounted, and observed by Leica light microscopy. Immunohistochemical staining was based on the percentage and intensity of the stained cells. Cells with immunoreactive cytoplasm (anti-Bax, anti-Bcl-2) were evaluated semiquantitatively (0: no staining; 1: 10–20; 2: 21–50; 3: 151–80, and 4: 180–100%) [20].

### Ethics Approval

This study was performed in line with the principles of the Declaration of Helsinki. Approval was granted by the Ethics Committee of University Mansoura faculty of Medicine (MS.21.12.1795). Informed consents were taken from all patients.

### Statistical Analysis

SPSS-PC software version 20 was used to perform all statistical calculations (MAS Medical and Scientific Eq. Co, IL, USA). The continuous data were expressed as mean ± standard deviation (SD) using independent-Sample *t* Test, as relevant. Categorical data, on the other hand, were represented as percentage and compared using Chi-square. Partial correlation coefficients (*r*) were calculated for the variables, after controlling for age and BMI.A *p* ≤ 0.05 was considered significant.

## Results

This study included 49 BC patients with mean age 61.93 ± 9.24 years.40 patients ((81.6%) were males and the rest 9 patients (18.4%) were females. In study group there were4 (8.2%) patients GI, 41 (83.7%) patients GII and 4 patients (8.2%) with undetected grade. Staging vary as following: 9 (18.4%) patients T1, 8 (16.3%) patients T2, 28 (57.1%) patients T3 and 4 (8.2%) patients T4 (Table 2).

**Table 2:** Clinical characteristics of patient

Character	No
no	49
Male n (%)	40 (81.6%)
Female n (%)	9 (18.4%)
Age (years) Mean ± SD	61.93 ± 9.24
BMI (kg/m2) Mean ± SD	29.89 ± 7.23
Hypertension n (%)	17 (34.7%)
Diabetes n (%)	11 (22.4%)
Smokingn (%)	6 (12.2%)
Grade n (%)	GII: 4 (8.2%) GIII: 41 (83.7%) Undetected grade: 4 (8.2%)
Stage n (%)	SI: 5(10.2%) SII: 5 (10.2%) SIII: 10 (20.4%) SIV: 4 (8.2%) Undetected stage: 25 (51%)
Stage T n (%)	T1: 9 (18.4%) T2: 8 (16.3%) T3: 28 (57.1%) T4: 4 (8.2%)
Stage N n (%)	N0: 32(65.3%) N1: 4(8.2%) N2:5 (10.2%) N3: 1 (2%) Undetected: 7 (14.3%)
Stage M n (%)	M0: 9 (18.9%) Mx: 25 (51%) Undetected: 15 (36.6%)

### Heavy Metals

In the analysis of heavy metals in cancer and non-cancerous tissues, Table 3 showed significant increase in Pb, Cr and Cd levels in cancer tissues than non-cancerous (*p* < 0.001). While Hg level was significantly decreased in cancer tissue (*p* < 0.001).

**Table 3:** Heavy metals between cancer and normal tissue

	cancer tissue	Non-canceousr tissue	*p* value
Pb (μg L^−1^) Median, (Range)	116.47 (13.76–393.44)	50.14 (10.58–87.54)	< 0.001
Cr (μg L^−1^) Median, (Range)	45.23 (13.76–364.34)	25.47 (8.53–46.32)	< 0.001
Hg (μg L^−1^) Median, (Range)	6.21 (3.11–25.63)	10.05 (0.46–57.33)	< 0.001
Cd (μg L^−1^) Median, (Range)	4.58 (0.83–12.16)	1.76 (0.01–3.78)	< 0.001

### Level of MDA and SOD Activity

Malondialdehyde (MDA) level was significantly increased in cancer tissue compared with non-cancerous tissue (*p* < 0.001). Superoxide dismutase (SOD) activity was significantly decreased in cancer tissue compared with non-canceroustissue (*p* < 0.001) (Table 4).

**Table 4:** MDA level and SOD activity in cancer and normal tissue

	Cancer tissue	non-canceousr tissue	*p* value
MDA (nmol ml^−1^) Median, (Range)	750.2 (172.02–1582.63)	100 (59.1–156.25)	< 0.001
SOD (U/gm) Mean ± SD	1767.59 ± 750.26	3180.10 ± 890.97	< 0.001

The correlation between heavy metals concentration and oxidative stress markers (Table 5) showed no significant correlation in both groups.

**Table 5:** Correlation coefficient (*r*) between blood levels of heavy metals with oxidative stress markers in cancer tissue and non-cancerous tissues

	MDA cancer tissue (nmol ml^−1^)		MDA non-cancerous tissue (nmol ml^−1^)		SOD cancer tissue (U/gm)		SOD non-cancerous tissue (U/gm)	
	*r*	*p*	*r*	*p*	*r*	*p*	*r*	*p*
Pb (µg L^−1^)	-0.040	0.783	-0.252	0.081	0.023	0.874	-0.035	0.812
Cr (µg L^−1^)	0.125	0.391	0.166	0.253	0.021	0.886	0.068	0.641
Hg (µg L^−1^)	0.121	0.408	0.025	0.862	-0.056	0.704	0.030	0.836
Cd (µg L^−1^)	-0.003	0.984	0.237	0.101	0.107	0.466	0.023	0.877

*r:* Correlation coefficient

### Gene Expression

In studding apoptotic pathway, Bcl2 expression was significantly increased in cancer tissue compared with non-cancerous tissue (*p* < 0.001). While Bax expression was significantly decreased in cancer tissues than non-cancerous tissues (*p* < 0.001) (Table 6).

**Table 6:** Gene expression in cancer and non-cancerous tissue

	Cancer tissue	non-cancerous tissue	*p* value
bcl2 Mean ± SD	4.01 ± 1.07	0.98 ± 0.079	< 0.001
Bax Mean ± SD	0.49 ± 0.23	0.99 ± 0.081	< 0.001
IL-6 Mean ± SD	2.96 ± 1.2	1.0 ± 0.05	< 0.001
AKT Mean ± SD	3.40 ± 1.73	1.02 ± 0.072	< 0.001
P38 Mean ± SD	3.76 ± 1.97	0.99 ± 0.055	< 0.001

The level of IL-6, AKT and P38 in bladder cancer tissues specimens were examined using RT-PCR. The results showed that cancer tissues expressed significantly higher levels of genes than non-cancerous bladder tissues (*p* < 0.001) (Table 6).

The correlation between heavy metals concentration and gene expression, Table 7 showed that in non-cancerous tissue there was no significant correlation between expression of studied genes and heavy metal concentrations (*p* > 0.05), while in cancer tissue, Cr level was significantly correlated with expression of Bax, AKT and P38 (*p* < 0.05). On the other hand, Cd level showed significant correlation with expression of Bax, IL-6, AKT and P38 (*p* < 0.001).

**Table 7:** Correlation coefficient (*r*) between blood levels of heavy metals with gene expression in adjacent non-cancerous tissue

	Bcl2		Bax		IL-6		AKT		P38	
	*r*	*p*	*r*	*p*	*r*	*p*	*r*	*p*	*r*	*p*
Pb (µg L-1)	0.068	0.645	0.014	0.924	0.215	0.137	-0.094	0.521	-0.005	0.97
Cr (µg L-1)	-0.125	0.394	-0.056	0.702	0.282	0.051	-0.247	0.086	0.052	0.721
Hg (µg L-1)	-0.01	0.943	0.108	0.459	-0.137	0.347	0.107	0.463	0.123	0.401
Cd (µg L-1)	0.005	0.975	0.075	0.608	0.093	0.526	-0.129	0.378	-0.018	0.902

### Immunohistochemistry

**Fig. 1:**
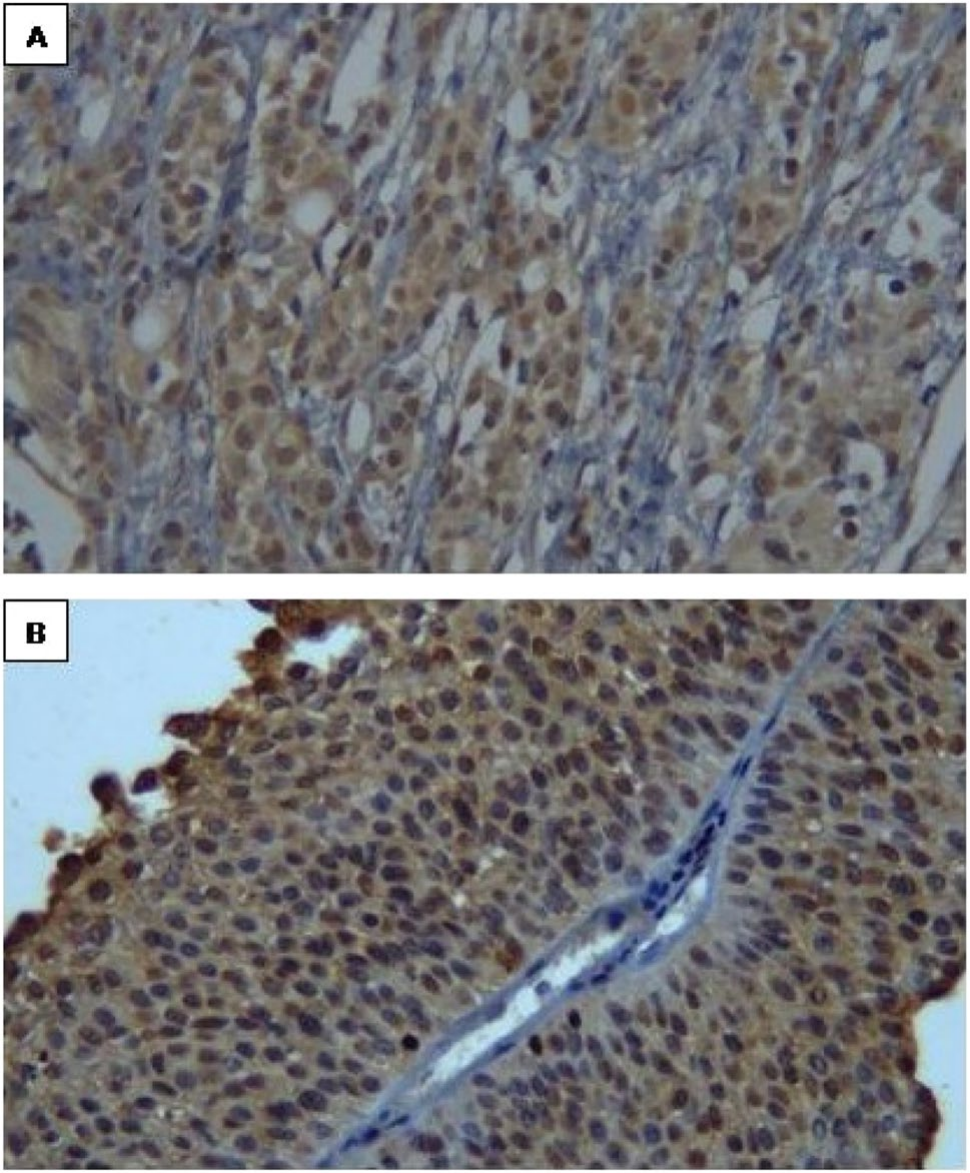
Representative examples of immunoreactivity for BCL2 showing: **a**) moderate expression in non-cancerous bladder tissues, and **b**) marked expression in cancer bladder tissues (× 200)

**Fig. 2:**
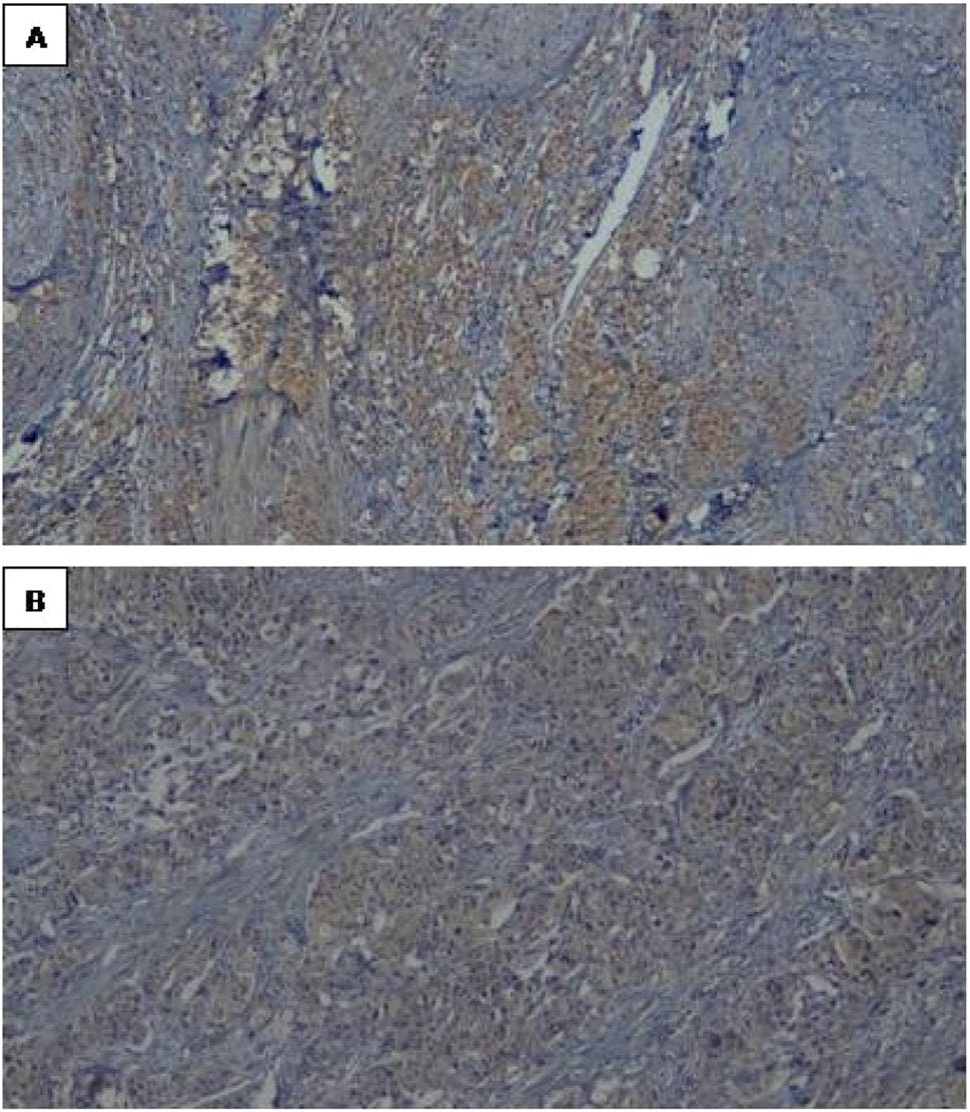
Representative examples of immunoreactivity for Bax showing: **a**) moderate expression in non-cancerous bladder tissues, and **b**) mild expression in cancer bladder tissues (× 100)

The protein expression of Bcl2 and Bax were detected in the 49 BC patients. Moderate expression of Bcl2 and Bax were observed in the non-cancerous tissues (Figs. 1A, 2A). In cancer tissue, Bcl2 showed marked expression, while Bax revealed mild expression (Figs. 1B, 2B). The H score for Bcl2 ranging from 10 to 285 (median: 160; interquartile range: 275) and 10–210 (median: 70; interquartile range: 200) for Bax. The expression of Bcl2 did not correlate with the levels of Pb, Cr, Hg, and Cd. In contrast, Bax expression was positively correlated with Cd levels (*r* = 0.357, *p* < 0.01).

## Discussion

Bladder cancer incidence is increasing nowadays due to occupational and environmental exposure. Exposure to heavy metal is reported to play contributory roles with increasing risk of BC [21]. Our study was conducted on 49 BC patient varying in their grades and stage in Urology and Nephrology Center, Mansoura University. Heavy metals concentrations were measured in cancer and non-cancerous tissue samples obtained from the patients. The result revealed that Pb, Cr and Cd levels were significantly increased in BC tissues compared to those in non-cancerous tissue, while a significant decrease in Hg level was observed in cancer tissue. These findings for Pb, Cd, and Cr which are in agreement with other studies [4, 22, 23] indicate that Pb, Cr, and Cd may have a role in the pathogenesis of BC.

Toxicity of some metal ions including Pb, Cr, Hg, and Cd stimulate oxidative stress, which leads to cellular damage in form of alteration in DNA and protein functions [24]. Malondialdehyde level is used as a marker of oxidative stress. We confirm in our study that cancer tissues showed significant increase in MDA level and a significant decrease in SOD activity compared with non-cancerous tissue of the same bladder. These results are confirmed by previous studies [25, 26]. İlhan et al. [27] reported that MDA level was significantly increased in tumor tissues of BC patients than in benign bladder tissue of the same patients while SOD activity decreased in bladder tumor tissues compared to non-cancerous bladder tissues. Also, Jeon et al. [28] stated that in BC patients, cancer tissues expressed SOD less than non-cancerous tissues.

Cancer is a genetic disorder caused by genetic mutations, which affect normal genes functions resulting in abnormal proteins. Therefore, gene expression assay can be used as a marker for cancer diagnosis, prognosis and progression. In this study, we investigated five genes using qRT-PCR [29]. Apoptosis is a key regulator in cancer pathway in response to DNA damage. Two groups of proteins of B-cell lymphoma-2 (Bcl-2) family regulate mitochondria apoptotic signaling: the first is anti-apoptotic proteins including Bcl-2 which suppress the release of cytochrome C and protect the mitochondrial outer membrane. The other is pro-apoptotic proteins including (Bax) which induce the release of cytochrome C and mitochondrial dysfunction which lead to apoptosis [11]. In the present study, Bcl2 expression was significantly increased in cancer tissues and Bax was significantly decreased in cancer tissues compared with non-cancerous tissue. Therefore, the overexpression of Bcl-2 has a key role in bladder cancer progression and aggressiveness [30]. Overexpression of Bax could be used as a prognostic marker for overall survival[31]. In a previous study,Gazzaniga et al. [32] reported similar results in bladder cancer tissues.

The present study showed that, the increase in Cr and Cd concentrations in cancer tissues compared to non-cancerous tissues is positively correlated with the expression of Bax gene with no significant correlation with Bcl2 expression. These results go along with that Cd exposure related to oxidative stress and apoptosis. Cr and Cd interact with Bax [33].

Our results showed that IL-6 was significantly expressed at higher levels in cancer tissues of BC than in non-cancerous tissues. In addition, we demonstrate a correlation between Cd level and gene expression of IL-6 in cancer tissue of bladder cancer. The relation between inflammation and cancer was described in previous studies, Interleukin-6 (IL-6), which is a cytokine that is involved in immune responses and inflammation and is known to be overexpressed in all tumor types. High levels of IL-6 promote tumorigenesis through regulating signaling pathways including metabolism, proliferation, invasiveness, angiogenesis, metastasis, apoptosis, and survival. Our results were confirmed by Chen et al. [34] who reported increased IL-6 expression in cancer tissue than non-cancerous tissue in BC patients. Previous study reported that exposure to some metal ions like Pb, Hg, and Cd linked to expression of IL-6 [35].

the expression of p38 in our study was significantly higher in cancer tissues than non-cancerous tissues of bladder cancer. In cancer tissues of bladder cancer, the expression of p38 was correlated with Cr and Cd levels. Our findings copy the previous studies reporting that expression of p38 in cancer tissues is higher than non-cancerous tissues of renal cell carcinoma [36]. Increased Cr and Cd levels induce oxidative stress and activate p38 MAP kinase pathway [37, 38].

P38 is member of mitogen-activated protein kinases (MAPKs) which mediated cell proliferation, migration, differentiation, invasion, and death [39]. p38 signaling is activated by variety of environmental stresses such as oxidative stress and toxicity with metal ions including Cd and Hg [40, 41]. In bladder cancer cells, activation of p38 related to tumor progression and metastasis [16].

AKT is a serine/threonine kinase also known as protein kinase B. It has a key role in regulating biological processes by phosphorylation of many enzymes, kinases and transcription factors. AKT promote cell survival and inhibits apoptosis by mediating the growth factors and inactivation of pro-apoptotic proteins. The hyperactivation of AKT is linked to metastasis BC [14, 15].Cr exposure induce activation of AKT pathway. Our results showed that AKT expression was significantly increased in cancer tissues of BC patients than non-cancerous bladder tissues and AKT expression significantly correlate with Cr and Cd. Whereas, Naji et al. [42] also found that Cd increase in the level of phosphorylated AKT in colon cancer.

## Conclusions

The study has confirmed the significant influence of Cr and cd but not Pb and Hg in bladder cancer progression through the expression of Bax, IL-6, AKT and p38 genes, while MDA and SOD as an oxidative stress markers did not show such influence. Further genetic studies may be required on the pathways in the process of cancer progression.

## Data Availability

All data and materials are available if requested.
